# Physiogenomic analysis of weight loss induced by dietary carbohydrate restriction

**DOI:** 10.1186/1743-7075-3-20

**Published:** 2006-05-15

**Authors:** Gualberto Ruaño, Andreas Windemuth, Mohan Kocherla, Theodore Holford, Maria Luz Fernandez, Cassandra E Forsythe, Richard J Wood, William J Kraemer, Jeff S Volek

**Affiliations:** 1 Genomas, Inc., 67 Jefferson St, Hartford, CT 06106, USA; 2 Department of Biostatistics, Yale University School of Medicine, New Haven, CT 06520, USA; 3 Department of Nutritional Sciences, University of Connecticut, Storrs, CT 06269, USA; 4 Human Performance Laboratory, Department of Kinesiology, University of Connecticut, Storrs, CT 06269, USA

## Abstract

**Background:**

Diets that restrict carbohydrate (CHO) have proven to be a successful dietary treatment of obesity for many people, but the degree of weight loss varies across individuals. The extent to which genetic factors associate with the magnitude of weight loss induced by CHO restriction is unknown. We examined associations among polymorphisms in candidate genes and weight loss in order to understand the physiological factors influencing body weight responses to CHO restriction.

**Methods:**

We screened for genetic associations with weight loss in 86 healthy adults who were instructed to restrict CHO to a level that induced a small level of ketosis (CHO ~10% of total energy). A total of 27 single nucleotide polymorphisms (SNPs) were selected from 15 candidate genes involved in fat digestion/metabolism, intracellular glucose metabolism, lipoprotein remodeling, and appetite regulation. Multiple linear regression was used to rank the SNPs according to probability of association, and the most significant associations were analyzed in greater detail.

**Results:**

Mean weight loss was 6.4 kg. SNPs in the gastric lipase (LIPF), hepatic glycogen synthase (GYS2), cholesteryl ester transfer protein (CETP) and galanin (GAL) genes were significantly associated with weight loss.

**Conclusion:**

A strong association between weight loss induced by dietary CHO restriction and variability in genes regulating fat digestion, hepatic glucose metabolism, intravascular lipoprotein remodeling, and appetite were detected. These discoveries could provide clues to important physiologic adaptations underlying the body mass response to CHO restriction.

## Introduction

A first line of attack on diabetes and cardiovascular disease is a reduction in body mass. A consistent finding across many different diet studies is that carbohydrate (CHO) restriction has a central role in facilitating weight loss and improving features of metabolic syndrome [1,2], due to mechanisms related to metabolic efficiency [3,4] and factors related to appetite regulation [5,6].

Genetic factors interact with dietary nutrients to impact the development of obesity and the outcome of weight loss therapies. The etiology of obesity is complex and can result from a disruption in functioning of diverse but interconnected pathways. Researchers have shown that polymorphisms in several different genes play a role in determining weight loss or weight maintenance in response to various pharmacological and non-pharmacological therapies. The approach taken in these genetic studies has been to separate individuals based upon allelic variation is a candidate gene and determine if weight loss or better maintenance of body mass varies as a function of the polymorphism. For example, body mass responses to various therapies have been linked to genes coding for products involved in the sympathetic nervous system [7-11], appetite regulating hormones [12,13], adipose tissue transcription factors [14,15], and proteins regulating fat digestion, deposition and mobilization [15-17].

In this study we use physiogenomics [18], a medical application of sensitivity analysis and systems engineering. Sensitivity analysis is the study of the relationship between input and output from a system as determined by each system component. Physiogenomics utilizes the genes as the components of the system. The gene variability, measured by single nucleotide polymorphisms (SNPs), is correlated to physiological responses of a population, the output. Physiogenomics determines how the SNP frequency varies among individuals similarly responding to the input over the entire range of the response distribution.

Scrutiny of weight loss responses for subjects who have consumed CHO restricted diets in our laboratory revealed a rather large amount of variability in the magnitude of weight loss [19,20]. This variability is not readily explained by standard covariates such as caloric intake, gender, age, activity, etc. Therefore a physiogenomic approach was undertaken using families of candidate genes, selected from those hypothesized to be involved in the metabolic adaptations induced by CHO restriction in the treatment of obesity. The results indicate that the magnitude of weight loss induced by a CHO restricted diet is, in part, explained by polymorphisms in specific genes among those we selected to study: genes that regulate lipases, intracellular glucose metabolism, HDL homeostasis, and appetite hormones.

## Methods

### Subjects and study design

The subjects included 86 adults who participated in very low CHO dietary studies designed to examine the effects on weight loss, body composition, and other metabolic responses related to cardiovascular disease in the Human Performance Laboratory at the University of Connecticut (Table 1). The subjects included 10 normal weight women studied over 4 weeks [21], 15 overweight men and 13 overweight women studied over 4–6 wk [20], 28 overweight men studied over 12 wk [22], and 10 overweight men and 10 overweight women with metabolic syndrome studied over 12 wk (unpublished). Subjects did not have diabetes, cardiovascular, respiratory, gastrointestinal, thyroid or any other metabolic disease. They were weight stable (± 2 kg) the month prior to starting the study, and were not allowed to use nutritional supplements (except a daily multi-vitamin/mineral), or be taking medications to control blood lipids or glucose. The majority of subjects were sedentary and all participants were instructed to maintain the same level of physical activity throughout the study. Before and after the low CHO diet, body mass was determined in the morning after an overnight fast on a calibrated digital scale with subjects in light clothing and not wearing shoes. All subjects signed an informed consent document approved by the Institutional Review Board.

**Table 1:** Mean body mass and weight loss broken down by gender, age, ethnicity, and length of diet.

**Factor**	**Level**	**N**	**Pre Body Mass (kg)**	**Change Body Mass (kg)**	**Genotyped**
All	All	86	89.4	-6.42	72
Gender	Female	33	74.7	-4.33	32
Gender	Male	53	98.5	-7.73	40
Age	<40 yr	56	88.8	-6.12	48
Age	40–49 yr	21	89.8	-6.08	16
Age	50–59 yr	6	94.7	-8.87	5
Age	60–69 yr	3	86.2	-9.70	3
Ethnicity	African Am	5	83.4	-5.60	3
Ethnicity	Asian	1	60.9	-2.30	1
Ethnicity	Caucasian	74	90.8	-6.52	63
Ethnicity	Hispanic	3	78.6	-6.10	3
Ethnicity	Indian	3	84.3	-7.13	2
Length	4	23	68.2	-2.20	23
Length	6	15	106.8	-6.27	11
Length	12	48	94.1	-8.50	38

### Dietary protocol

The diet intervention was free-living with the main goal to restrict CHO to a level that induced a small level of ketosis. There were no restrictions on the type of fat from saturated and unsaturated sources or cholesterol levels. Foods commonly consumed were beef (e.g., hamburger, steak), poultry (e.g., chicken, turkey), fish, vegetable oils, various nuts/seeds and peanut butter, moderate amounts of vegetables, salads with low CHO dressing, moderate amounts of cheese, eggs, protein drinks, and water or low CHO diet drinks. The use of sugar alcohol-containing low CHO foods was permitted but limited to one item per day. To ensure appropriate CHO restriction, subjects monitored their level of ketosis daily using urine reagent strips that produce a relative color change in the presence of one of the primary ketones, acetoacetic acid. We have found this to be a very sensitive indicator of CHO restriction and compliance. Blood ketones were also checked during the diets. On this basis, all subjects in our low CHO studies were in ketosis for the majority of the experimental period. All subjects received extensive initial verbal and written instructions and weekly follow-up dietetic education. Subjects received thorough instructions for completing detailed weighed food records during baseline and various phases of the diet that were subsequently analyzed using regularly updated nutrient analysis software. The actual mean nutrient breakdown of the diets as a percentage of total energy as obtained from at least 15 days of weighed food records from four cohorts of subjects was 8–13% CHO, 60–63% fat, and 28–30% protein (see Supplemental File).

### Candidate gene selection

Eleven candidate genes were broadly selected for their various roles in regulation of body weight. We chose representative genes coding for products from four pathways and processes that we hypothesized as playing an important role in mediating weight loss induced by CHO restriction including (1) enzymes regulating digestion, trafficking, and intracellular metabolism of fat, (2) enzymes regulating intracellular glucose metabolism, (3) proteins affecting lipoprotein remodeling and metabolism, and (4) hormones regulating appetite (Table 2).

**Table 2:** List of 15 genes and 27 SNPs studied for association with weight loss induced by a low CHO diet.

**Gene Family or Pathway**	**Gene**	**SNP**	**N**	**mac**	**min**	**maj**	**Freq**	**Gene**	**Description**	**Sequence Context**
Lipases	Hepatic lipase	rs936960	49	7	A	C	0.07	LIPC	lipase, hepatic	CAGAGCACGAGGCTGATTTTC [A/C]ATCCCAGTGTGGGCCACACC
		rs417344	50	13	T	C	0.13	LIPC	lipase, hepatic	TTTCCTAATTTTGCAGTTGAG [A/G]TTTAAGAGGTTGGGAACTGG
		rs6083	39	28	A	G	0.36	LIPC	lipase, hepatic	GTCTTTCTCCAGATGATGCCA [A/G]TTTTGTGGATGCCATTCATA
	Lipoprotein lipase	rs295	46	15	A	C	0.16	LPL	lipoprotein lipase	GATGCACCTACTAGACACCTA [A/C]TCTGCGCTAGATGGTGGGGG
		rs328	53	10	C	G	0.09	LPL	lipoprotein lipase	ACAAGTCTCTGAATAAGAAGT [C/G]AGGCTGGTGAGCATTCTGGG
	Hormone sensitive lipase	rs10422283	43	26	T	C	0.30	LIPE	lipase, hormone-sensitive	GGAAGGAACCTCGTACATCCT [A/G]CGGGGCAGTGGGGACAGCGT
	Lysosomal acid lipase	rs1556478	35	28	A	G	0.40	LIPA	lipase A, lysosomal acid, cholesterol esterase (Wolman disease)	CACGGAGACTTATGCACCAGA [A/G]TGAAATGCTGAGATGTTCTT
		rs6586179	45	7	T	C	0.08	LIPA	lipase A, lysosomal acid, cholesterol esterase (Wolman disease)	ACCCTGCATTCTGAGGGGTCT [A/G]GAGGGAAACTGACAGCTGTG
	Endothelial lipase	rs4245232	45	15	A	C	0.17	LIPG	lipase, endothelial	TAAAAAACTAAAGCCCGCCTG [A/C]GTCTTGTTAATGAATGATAG
	Gastric lipase	rs814628	45	9	A	G	0.10	LIPF	lipase, gastric	ATCGACTTCATTGTAAAGAAA [A/G]CTGGACAGAAGCAGCTACAC
Glycogen Synthases	Glycogen Synthase 1 (muscle)	rs2287754	35	16	A	G	0.23	GYS1	glycogen synthase 1 (muscle)	CGGGAAGCTTGCAAGACGCTC [A/G]GCTTCCTATTGCAAGACCGC
	Glycogen Synthase 2 (hepatic)	rs1478290	59	29	T	G	0.25	GYS2	glycogen synthase 2 (liver)	AATGTGGCTGAAGCCAAAAGC [A/C]TAATGAATGAGGGGAAGCCT
		rs1871143	40	23	T	G	0.29	GYS2	glycogen synthase 2 (liver)	AGCCAGGAGCTTTCCTGGGCG [A/C]TTTTTGTACAGGATCTCATT
		rs2306179	44	18	A	G	0.20	GYS2	glycogen synthase 2 (liver)	TTTCAGTAGGTTTGCAGGGAA [A/G]CCAACTCAAAGCTATATCTG
	Glycogen Synthase 3b	rs4688046	44	19	T	C	0.22	GSK3B	glycogen synthase kinase 3 beta	TAGTAAACTATTTCTTCCCAT [A/G]GGAGAAGATGGATTCTTTTC
		rs334555	43	7	C	G	0.08	GSK3B	glycogen synthase kinase 3 beta	AATTATATCTTATTATTAAAA [C/G]TCTACCAACTCAAAGCTTCC
HDL Homeostasis	CETP	rs711752	46	36	A	G	0.39	CETP	cholesteryl ester transfer protein, plasma	TTCAAGGTCAAGTTCTTTGGT [A/G]AGAAGGTCCTAGCTGCATTG
		rs3764261	41	20	T	G	0.24	CETP	cholesteryl ester transfer protein, plasma	AGTGAATGAGATAGCAGACAA [A/C]CCAGATGCCTACCGACAGGT
		rs5880	44	4	C	G	0.05	CETP	cholesteryl ester transfer protein, plasma	GATATCGTGACTACCGTCCAG [C/G]CCTCCTATTCTAAGAAAAGC
		rs1532624	51	33	T	G	0.32	CETP	cholesteryl ester transfer protein, plasma	TCTGCCCCTTTGGGCTGCAGC [A/C]TCACAAGCTGTGTGGCGTTG
		rs5883	56	8	T	C	0.07	CETP	cholesteryl ester transfer protein, plasma	AGCTACCTTGGCCAGCGAGTG [A/G]AAGACTCGCTCAGAGAACCA
	APOA1	rs5070	41	18	A	G	0.22	APOA1	apolipoprotein A-I	GCCACGGGGATTTAGGGAGAA [A/G]GCCCCCCGATGGTTGGCTCC
	APOC3	rs4520	38	23	T	C	0.30	APOC3	apolipoprotein C-III	CTTGGTGGCGTGCTTCATGTA [A/G]CCCTGCATGAAGCTGAGAAG
		rs2071521	45	37	T	C	0.41	APOC3	apolipoprotein C-III	ACAGCTCCTGTTGCCATAGGA [A/G]GGAGCTGGGTGAGATACTAG
Appetite Hormones	Galanin	rs694066	56	6	A	G	0.05	GAL	galanin	TTCTAAGTCCTCTGCCATGCC [A/G]GGAAAGCCTGGGTGCACCCA
	Neuro-peptide Y	rs1468271	48	5	A	G	0.05	NPY	neuropeptide Y	GACCCTGTAATTTTCAGAAAC [A/G]CACATAGGAGTGGGTGTCTG
	Ghrelin Precursor	rs26312	63	14	A	G	0.11	GHRL	ghrelin precursor	GCTGTTGCTGCTCTGGCCTCT [A/G]TGAGCCCCGGGAGTCCGCAG

SNP identification numbers (noted as "rs...") are the unique SNP identifiers from the NCBI dbSNP database. Also given are the number of patients with good genotype results (N), the number of minor alleles found (mac), and the corresponding allele frequency as observed in this study.

### Laboratory analysis

Blood samples were collected from an arm vein into tubes for DNA extraction. The DNA was extracted from 8.5 mL of whole blood using the PreAnalytiX PAXgene DNA isolation kit (Qiagen Inc, Valencia, CA). For some earlier participants, neither whole blood nor DNA were available, so DNA from lymphocytes remaining in archived serum samples were amplified using the QiaGen REPLI-g Whole Genome Amplification kit. Genotyping was performed using the Illumina BeadArray™ platform and the GoldenGate™ assay [23,24]. The assay information and observed allele frequencies for the SNPs used in this study are listed in Table 2. Genotype calls of sufficient quality could not be obtained for 14 subjects, which were left in the study to contribute to the covariate model, but did not contribute directly to the genetic associations.

### Data analysis

All statistical analysis was performed using the R Statistics Language and Environment [25-27]. Covariates were analyzed using multiple linear regression, and selected using the stepwise procedure. To test for association with SNP genotypes, the residual of Δbody mass from the covariate model was tested using linear regression on the SNP genotypes. SNP genotype was coded quantitatively as a numerical variable indicating the number of minor alleles: 0 for major homozygotes, 1 for heterozygotes, and 2 for minor homozygotes. The F-statistic p-value for the SNP variable was used to evaluate the significance of association. To test the validity of the p-values, we also performed an independent calculation of the p-values using permutation testing. The ranking of the first three SNPs were identical under permutation and F-statistic analyses (data not shown). To account for the multiple testing of 27 SNPs, we calculated adjusted p-values using Benjamini and Hochbergs false discovery rate (FDR) procedure [28-30]. In addition, we evaluated the power for detecting an association based on the Bonferroni multiple comparison adjustment. We calculated for each SNP the effect size in standard deviations that is necessary for detection of an association at a power of 80% (20% false negative rate) using the formula



where a is the desired false positive rate (a = 0.05), b the false negative rate (b = 1-Power = 0.2), c the number of SNPs, z a standard normal deviate, N the number of subjects, f the carrier proportion, and Δ the difference in Δbody mass between carriers and non-carriers expressed relative to the standard deviation [31].

### LOESS representation

We use a locally smoothed function of the SNP frequency as it varies with body mass to visually represent the nature of an association. LOESS (LOcally wEighted Scatter plot Smooth) is a method to smooth data using a locally weighted linear regression [32,33]. At each point in the LOESS curve, a quadratic polynomial is fitted to the data in the vicinity of that point. The data are weighted such that they contribute less if they are further away, according to the tricubic function



where *x *is the abscissa of the point to be estimated, the *x*_*i *_are the data points in the vicinity, and *d(x) *is the maximum distance of *x *to the *x*_*i*_.

## Results

**Figure 1:**
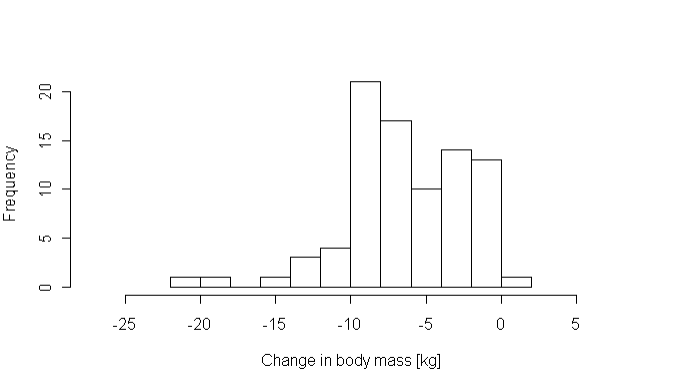
Distribution of change in body mass (weight loss) in the study population.

Table 1 summarizes the weight loss data available for the study population. The distribution of weight loss was non-gaussian (Figure 1). Of the potential covariates listed in Table 3, length of diet, body mass index, and baseline total cholesterol were significantly associated with weight loss. Length of diet, in particular, accounted for an increased weight loss of 0.55 kg with each additional week of diet. Increased baseline body mass index (BMI) was also correlated with weight loss. There was a relationship between total cholesterol level and weight loss, with an additional 19 grams of weight lost for every mg/dl of total cholesterol at the beginning of the study.

**Table 3:** Potential covariates examined. The 3 statistically significant ones are shown in italics with corresponding significance level, p value.

**Name**	**Measure**	**Description**	**p-value**
Gender	male, female	Patient gender	
Age	integer	Patient age	
Ethnicity	African American, Asian, Caucasian, Hispanic, Indian	Patient self reported ethnicity	
*Length*	4, 6, or 12 wk	*Length of diet*	1.00E-12
*TC*	mg/dl	*Total cholesterol*	0.04
LDL	mg/dl	LDL cholesterol	
HDL	mg/dl	HDL cholesterol	
TG	mg/dl	Tryglycerides	
THDLR	ratio	Ratio of Total to HDL-C	
BMS	kg	Body mass	
FM	kg	Fat mass	
LBM	kg	Lean body mass	
PF	percent	Percent body fat	
*BMI*	kg/m2	*Body mass index*	3.40E-06

After adjusting for the associations in Table 3, each SNP in Table 2 was tested for association with the residual variable (body mass adjusted for the covariates) (Table 4). Of the 27 SNPs, four had a statistically significant association with the residual body mass, namely gastric lipase (LIPF, SNP rs814628), cholesteryl ester transfer protein (CETP, SNP rs5883), hepatic glycogen synthase 2 (GYS2, SNP rs2306179), and galanin (GAL, SNP rs694066). The first three results remained significant when adjustment was made for the testing of multiple SNPs using the FDR method.

**Table 4:** Results of the association test of each SNP against the phenotypic variation residual from the regression of covariates in Table 3. Results significant at alpha ≤ 0.05 are indicated in bold. Also shown are the FDR corrected p-value, the degrees of freedom in the regression model, the regression coefficient indicating the size of the effect of the minor allele, and the minimum effect size for 80% power of detection, relative to the standard deviation.

**SNP**	**Gene**	**p-value**	**FDR**	**degf**	**coeff**	**power**	**SNP type**
rs936960	LIPC	0.7354	0.8011	47	0.351	2.08	intron 1
rs417344	LIPC	0.1322	0.5433	48	1.175	1.57	~5.5 kb upstream from LIPC
rs6083	LIPC	0.6944	0.8011	37	-0.226	1.25	S215N
rs295	LPL	0.5519	0.7843	44	-0.526	1.49	intron 6
rs328	LPL	0.1973	0.5744	51	-1.330	1.76	exon 9, *474S
rs10422283	LIPE	0.1610	0.5433	41	0.916	1.24	intron 1
rs1556478	LIPA	0.7417	0.8011	33	0.228	1.29	intron 5
rs6586179	LIPA	0.2575	0.6321	43	-1.189	2.08	exon 1, R23G
rs4245232	LIPG	0.1561	0.5433	43	0.906	1.50	~1.5 kb upstream
**rs814628**	**LIPF**	**0.0002**	**0.0059**	**43**	**-3.658**	**1.86**	**exon 4, Ala161>Thr**
rs2287754	GYS1	0.6950	0.8011	33	-0.324	1.51	5' UTR
rs1478290	GYS2	0.4504	0.7843	57	-0.381	1.13	~3.5 Kb upstream
rs1871143	GYS2	0.5236	0.7843	38	-0.381	1.31	intron 11
**rs2306179**	**GYS2**	**0.0068**	**0.0610**	**42**	**-1.717**	**1.40**	**intron 5**
rs4688046	GSK3B	0.5148	0.7843	42	-0.382	1.37	intron 3
rs334555	GSK3B	0.4553	0.7843	41	0.925	2.09	intron 1
rs711752	CETP	0.2127	0.5744	44	0.677	1.13	intron 1
rs3764261	CETP	0.6900	0.8011	39	0.263	1.36	~2.6 kb upstream
rs5880	CETP	0.0782	0.4220	42	2.769	2.71	P390A
rs1532624	CETP	0.5299	0.7843	49	0.339	1.12	intron 7
**rs5883**	**CETP**	**0.0018**	**0.0237**	**54**	**-2.854**	**1.94**	**exon 9, synonymous**
rs5070	APOA1	0.4406	0.7843	39	-0.454	1.41	Intron
rs4520	APOC3	0.4275	0.7843	36	-0.497	1.32	G34G
rs2071521	APOC3	0.9176	0.9176	43	-0.053	1.13	Upstream
**rs694066**	**GAL**	**0.0231**	0.1557	**54**	**2.105**	**2.22**	**intron 1**
rs1468271	NPY	0.7401	0.8011	46	-0.378	2.43	intron 1
rs26312	GHRL	0.7832	0.8133	61	0.233	1.50	~1 kb upstream

**Figure 2:**
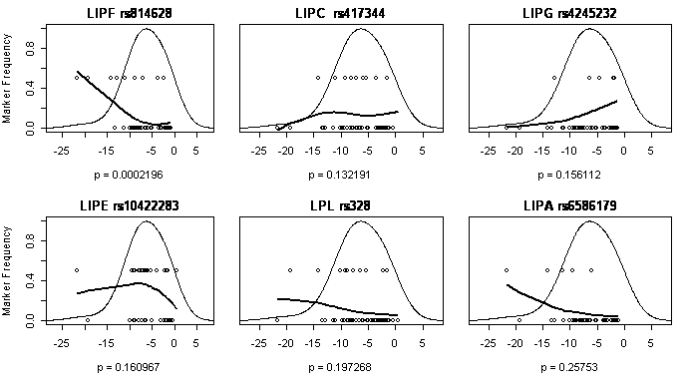
LOESS plots for six lipase genes listed in order of genetic association significance, as follows: LIPF gastric lipase, LIPC hepatic lipase, LIPG endothelial lipase, LIPE hormone-sensitive lipase, LPL lipoprotein lipase, LIPA lipase A lysosomal acid. One SNP is shown per gene, with corresponding significance level (p value), from Table 4. SNP rs814628 of the gastric lipase (LIPF) gene was significantly associated with weight loss while the others were not. The x-axis is the same as in figure 1: change in body mass [kg].

**Figure 3:**
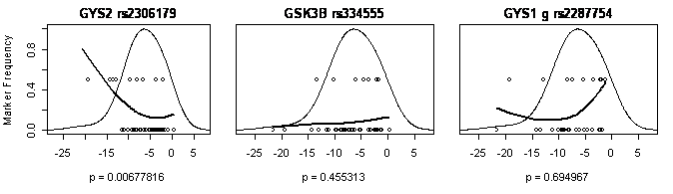
LOESS plots for 3 glycogen synthase genes listed in order of genetic association significance, as follows: GYS2 glycogen synthase 2 (liver), GSK3B glycogen synthase kinase 3 beta, GYS1 glycogen synthase 1 (muscle). One SNP is shown per gene, with corresponding significance level (p value), from Table 4. SNP rs2306179 of the GYS2 gene was significantly associated with weight loss while the others were not. The x-axis is the same as in figure 1: change in body mass [kg].

**Figure 4:**
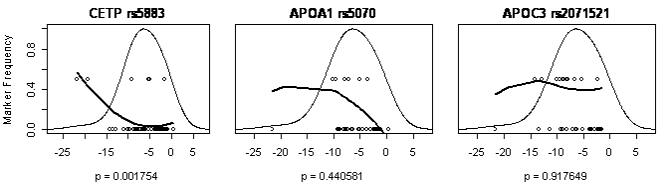
LOESS plots for the lipid metabolism listed in order of genetic association significance, as follows: CETP, cholesteryl ester transfer protein, plasma, APOA1 apolipoprotein A-I, APOC3 apolipoprotein C-III. One SNP is shown per gene, with corresponding significance level (p value), from Table 4. SNP rs5883 of the CETP gene was significantly associated with weight loss while the others were not. The x-axis is the same as in figure 1: change in body mass [kg].

**Figure 5:**
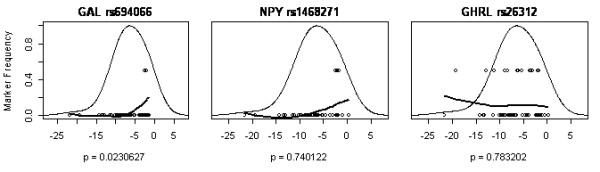
LOESS plots for the appetite regulation hormone listed in order of genetic association significance, as follows: GAL galanin, NPY neuropeptide Y, GHRL ghrelin precursor. One SNP is shown per gene, with corresponding significance level (p value), from Table 4. SNP rs694066 of the GAL gene was significantly associated with weight loss while the others were not.

Figures 2, 3, 4, 5 show a detailed representation of the genetic association tests for all genes. The overall distribution of change in body mass is shown along with the individual genotypes and a LOESS fit of the allele frequency as a function of body mass. The bell curve shows the fitted distribution of body mass phenotype in the clinical studies. The LOESS curve shows the localized frequency of the least common allele for sectors of the distribution. For SNPs with a strong association, the marker frequency will be significantly different between the high end and the low end of the distribution. Conversely, if a marker is neutral, the frequency will be independent on the body mass and the LOESS curve will be essentially flat.

For example, the first panel in Figure 2 shows the LOESS curve for SNP rs814628, which is located in the gene for gastric lipase (LIPF). The frequency of the minor allele approaches 60% at the highest amount of weight loss (left of the distribution) whereas it is 5% in subjects with little body mass change (right of the distribution). The overall frequency of this SNP in the study population is 10% (Table 2). This marked dependence of SNP frequency with the phenotype is indicative of a strong association between the gene marker and body mass, as attested by a p value of 0.0002 (Table 4).

## Discussion

The principal metabolic adaptations contributing to weight loss induced by CHO restriction are unknown. This study used physiogenomic analysis to examine the relations between genes regulating target proteins impacting the intake and metabolism of dietary nutrients and weight loss in subjects on a very low CHO diet. The change with length of diet is a good confirmation that it is indeed the low CHO intervention that caused the weight loss, although it is unknown if similar changes would be found if weight loss were brought about by other types of diets. Genetic polymorphisms also significantly associate with weight loss when these factors are used as a covariate indicating that genetic variation may be a valuable tool to predict individual variability in weight loss to dietary CHO restriction. The results indicate that common genetic markers in gastric lipase, glycogen synthase, CETP, and galanin have a substantial effect on weight loss response to CHO restricted diets.

Very low CHO diets are typically higher in fat. We therefore hypothesized that polymorphisms in various lipases may account for variability in weight loss. We tested SNPs in genes for gastric, hepatic, lipoprotein, hormone-sensitive, lysosomal acid, and endothelial lipase. Unexpectedly, we discovered the gastric isoenzyme was the most significant genetic association to weight loss, whereas other lipases were not. Gastric lipase is secreted by the mucosa of the stomach and hydrolyzes the ester bonds of dietary triglycerides in the gastrointestinal tract. Pancreatic lipase is generally the dominant enzyme in the hydrolysis of gastrointestinal lipids, but gastric lipase can contribute significantly especially in instances where pancreatic lipase is deficient [34]. Thus a significant locus of variability is the ability to perform first pass breakdown of dietary fat. Individuals with the least common version of the enzyme had the most weight loss, indicating that impairment of gastric fat breakdown on a low CHO diet enhances weight loss.

The second category of genes we surveyed was related to glycogen synthesis. Glycogen synthase catalyzes the formation of glycogen from glucose. A defect in this pathway in skeletal muscle has a dominant role in the insulin resistance that occurs in diabetes [35]. Polymorphisms in glycogen synthase kinase beta (GSK3B), a regulator of glycogen synthase activity, and skeletal muscle glycogen synthase 1 (GYS1) have been examined in several studies, but have generally failed to associate with diabetes or measures of insulin resistance [36,37]. In addition to GSK3B and GYS1, we examined hepatic glycogen synthase 2 because recent work also indicates that insulin stimulated hepatic glycogen synthesis is impaired in diabetics [38]. We discovered that a polymorphism in hepatic, but not skeletal muscle, glycogen synthase was associated with weight loss. The results suggest that the hepatic response to carbohydrate restriction may influence the weight loss response to a low CHO diet.

In addition to weight loss, our prior work has shown that low CHO diets result in reliable and dramatic changes in lipoprotein metabolism characterized by decreased triglycerides and remodeling of LDL and HDL cholesterol to form larger particles [1,22]. Since weight loss has similar effects, we surveyed various apolipoproteins and enzymes regulating triglyceride and lipoprotein metabolism including CETP, apolipoprotein A-I, and apolipoprotein C-III. We found that a specific polymorphism in plasma CETP was significantly associated with weight loss. The major function of CETP is the net mass transfer of cholesterol esters from HDL to triglyceride-rich lipoproteins and LDL-C and of triglyceride-rich lipoproteins to HDL-C and LDL-C [39], thereby providing a mechanistic link to explain the triglyceride lowering and remodeling effects of LDL and HDL observed with low CHO diets [22,40]. Several studies have linked polymorphisms in the CETP gene to lipoprotein responses and risk for cardiovascular disease, and it has been hypothesized that these relations may be altered by weight loss [41]. Our study is the first study to show an association of a polymorphism in CETP gene to weight loss. The finding suggests that the weight loss response to CHO restriction may be mechanistically linked to the intravascular processing of lipoproteins.

Hormonal regulation of food intake was hypothesized to be one mechanism by which CHO restricted diets affect weight loss. We examined polymorphisms in galanin, neuropeptide Y, and ghrelin. Galanin was the only hormone significantly associated with weight loss. Galanin stimulates food consumption, particularly fat intake. A prior study that measured polymorphisms in galanin failed to find an association with fat intake or obesity [42]. Prior work has shown that galanin in the para-ventricular nucleus is stimulated by a fat feeding and increased circulating triglycerides, which in turn promotes further fat consumption in a non-homeostatic feed-forward manner [43]. The finding in this study that a polymorphism surveyed in the galanin gene was associated with weight loss provides evidence for a role of fat-mediated appetite hormones in determining the response to carbohydrate restriction.

Physiogenomics introduces a new paradigm in the genetic analysis of complex phenotypes. Historically, a candidate gene approach identified one specific hypothesis. However, such focused hypotheses are often unrealistic given the number of overlapping pathways at the organismic, cellular, and molecular levels. Array technologies provide efficient methods to simultaneously probe large numbers of genes using general hypotheses about entire pathways and systems. As a previous example of this approach, we had demonstrated a strong association between CK activity during statin treatment and variability in genes related to vascular function, angiotensin II Type 1 receptor (AGTR1) and nitric oxide synthase 3 (NOS3) [44]. This finding had led us to suggest the novel hypothesis that vascular smooth muscle function may contribute to the muscle side effects of statins.

Similarly, we believe that novel hypotheses have been generated in this study. The approach to select gene families or functionally related genes generates positive and negative results for physiogenomic analysis. It is the contrast in statistical significance levels within each of the four functional groups pursued in this study that provides the mechanistic insight. The associated gene markers can be combined into SNP ensembles harnessing their combined predictive power to estimate weight loss attainable from carbohydrate restriction for each individual. The SNP ensemble can then be tested retrospectively or prospectively to assess its predictive diagnostic power in populations separate from the ones used to generate the model. We believe this approach is pivotal to the discovery of multi-gene effects determining human dietary response. Applications to the management of obesity and diabetes include individualized counseling and dietary choice based on innate capacity to react to various nutritional regimens. We foresee the translation of these findings to diagnostic systems for personalized diet.

## Competing interests

Dr. Ruano, Mr. Kocherla and Dr. Windemuth are full-time employees and shareholders of Genomas Inc.

## Authors' contributions

JSV, MLF, WJK were involved with the acquisition of funding, design, conception and execution of the dietary intervention and biochemical analysis portion of the study, as well as acquisition, analysis and interpretation of data. CEF and RJW were primarily involved in data acquisition, analysis and interpretation of data. GR, AW, and MK performed the genetic analysis. GR, TH, and AW performed the statistical analysis. All authors were involved in the drafting and revision of the manuscript and give their final approval of this version to be published.
